# A Novel Adaptive Terminal Sliding Mode Tracking Control of Engine Electronic Throttle System Using an Enhanced Observer Under Composite Disturbances

**DOI:** 10.3390/s26196070

**Published:** 2026-09-25

**Authors:** Van Du Phan, Van Chuong Le, Van Nam Dinh, Dinh Tu Duong, Sy Phuong Ho

**Affiliations:** College of Engineering and Technology, Vinh University, Vinh City 43100, Vietnam

**Keywords:** adaptive terminal sliding mode control, enhanced observer, disturbance estimation, finite-time convergence

## Abstract

This paper proposes a novel adaptive terminal sliding mode tracking control (ATSMC) strategy integrated with an enhanced super-twisting extended state observer (ESESO) for an engine electronic throttle system (EETS) operating under composite disturbances. First, a nonlinear dynamic model of the ETS is established by considering actuator nonlinearities, spring torque effects, friction uncertainties, and external load disturbances. Subsequently, an enhanced observer is developed to accurately estimate the lumped disturbance composed of model uncertainties, unmodeled dynamics, and external perturbations in real time. Based on the observer output, an adaptive terminal sliding mode controller is designed to achieve fast finite-time convergence while eliminating the requirement for prior knowledge of disturbance bounds. An adaptive law is introduced to automatically adjust the control gain, thereby reducing conservatism and mitigating chattering phenomena. Lyapunov stability analysis is conducted to rigorously prove the finite-time convergence and boundedness of all closed-loop signals. Comparative experimental studies demonstrate that the proposed method achieves superior tracking accuracy compared with conventional sliding mode and adaptive control approaches.

## 1. Introduction

The engine electronic throttle system (EETS) has become a key component in modern automotive engine management systems due to its capability to precisely regulate the air intake flow and improve engine performance, fuel economy, emission control, and advanced driver assistance systems [1,2,3]. Despite its advantages, achieving high-precision throttle position control remains a challenging task because the EETSs exhibits strong nonlinear characteristics and is subject to various uncertainties. In practice, the system dynamics are influenced by spring nonlinearities, friction effects, actuator parameter variations, sensor imperfections, external load torques, and unmodeled dynamics [4,5]. These factors can be collectively represented as lumped disturbances, which significantly degrade tracking accuracy, increase transient oscillations, and may even compromise system stability if not properly compensated [6,7]. Furthermore, the magnitude and characteristics of these disturbances vary with operating conditions, making their accurate modeling and compensation particularly difficult [8]. Therefore, developing a new control framework that simultaneously improves tracking performance, disturbance rejection capability, and control smoothness despite complex operating conditions is essential.

To address these challenges, numerous control approaches have been investigated for EETS applications, including proportional-integral-derivative (PID) control [9], sliding mode control (SMC) [10,11], adaptive control [12,13], intelligent control [14], neural-network-based control [15], and disturbance-observer-based control techniques [16,17]. Among these methods, sliding mode control has attracted considerable attention due to its inherent robustness against matched uncertainties and lumped disturbances [18]. However, conventional sliding mode controllers often suffer from chattering phenomena and may exhibit degraded performance when disturbance bounds are unknown [19,20]. Adaptive control techniques have been introduced to improve parameter adaptation capabilities, while terminal SMC (TSMC) has been employed to achieve faster convergence and higher tracking accuracy [21,22]. In [23], the authors presented an adaptive integral TSMC strategy integrated with an uncertainty observer for EETSs to achieve high-precision angle throttle tracking performance. In [24,25], an extreme learning machine (ELM)-based robust TSMC strategy was proposed for automotive EETSs with uncertain dynamics. In [26], continuous fast nonsingular TSMC was introduced to tackle the effects of multiple nonlinearities for EETSs. However, the controller contains multiple design parameters associated with the terminal sliding surface, observer gains, and continuous switching function. Proper tuning is required to achieve the desired performance, yet systematic tuning guidelines may not be available.

In recent years, observer-based control strategies have emerged as an effective solution for enhancing disturbance rejection performance [27,28,29]. By estimating lumped disturbances in real time and compensating for them through feedforward mechanisms, disturbance observers can significantly improve tracking precision without requiring an accurate system model. In particular, enhanced state observers are capable of providing fast and accurate disturbance estimation while suppressing noise amplification, making them suitable for automotive actuator applications [30]. In [31], the author presented an extended state observer (ESO)-based adaptive TSMC scheme for automobile EETSs to enhance control performance under lumped uncertainty. In [32], an adaptive second-order fixed-time strategy integrated disturbance rejection for the EETSs subject to lumped disturbances was suggested. In [33], a continuous predefined-time disturbance observer-based ASMC was developed for EETSs with effectiveness validated through simulations. In [34], a high-precision angle regulation-based observer and discrete-time SMC strategy for EETSs in the presence of nonlinearities and disturbances was reported to improve disturbance rejection performance. Nevertheless, the observer gains and controller parameters must be appropriately tuned, and achieving finite-time convergence does not necessarily imply optimal tracking accuracy or robustness in the presence of practical uncertainties [35,36,37,38].

Motivated by these considerations, this work investigates the novel trajectory tracking control problem of EETSs under lumped disturbances. The proposed control framework aims to improve the overall performance of the ETS while maintaining smooth control action under complex operating conditions. The main contributions can be summarized as follows:

1. An enhanced super-twisting extended state observer (ESESO) is constructed to estimate the composite disturbances arising from model uncertainties, friction nonlinearities, and external perturbations. The observer provides real-time disturbance information to improve the disturbance rejection capability.
2. A novel adaptive terminal sliding mode control (ATSMC) strategy is developed to adjust the control gain online and achieve fast and accurate throttle angle tracking. The terminal sliding manifold ensures finite-time convergence of the tracking error, thereby improving transient and steady-state performance.
3. Extensive experimental studies are performed on an EETS test bench, and the proposed framework effectively compensates for composite disturbances and improves tracking accuracy compared with existing control methods.

## 2. Description and Mathematical Modeling

The EETS is an electromechanical actuator that regulates the airflow entering the engine by controlling the throttle plate angle. The system typically consists of a DC motor, reduction gears, return springs, friction mechanisms, and a throttle position sensor as shown in Figure 1. Considering nonlinear spring characteristics, friction effects, and external disturbances, the ETS exhibits highly nonlinear dynamic behavior. Considering the electrical and mechanical dynamics, the armature circuit of the DC motor is described by:

(1) $$\dot{i}=-\frac{K_{e}}{L}\omega -\frac{R}{L}i+\frac{1}{L}u$$

where u denotes the control voltage, R, *i* and L are the armature resistance, armature current, and inductance, respectively, $K_{e}$ is the back-EMF constant, and ω is the motor angular velocity.

Since the electrical dynamics are significantly faster than the mechanical dynamics, the armature inductance is often neglected, and the electromagnetic torque of the motor is expressed as

(2) $$i=\frac{u-K_{e}\omega }{R};T_{m}=K_{t}i=K_{t}\frac{u-K_{e}\omega }{R}$$

where $K_{t}$ is the motor torque constant.

The rotational dynamics of the throttle plate can be formulated by Newton’s second law:

(3) $$J\ddot{ο}=T_{m}-T_{s}-T_{f}sgn(\omega )-B\omega -T_{L},$$

where J and ο define the equivalent rotational inertia and throttle angle; *B* denotes the viscous damping coefficient; $T_{s},T_{f},\;\mathrm{and}\;T_{L}$ define the spring torque, the friction torque, and external load disturbances and unmodeled dynamics.

Combining the electrical and mechanical dynamics yields the following:

(4) $$J\ddot{ο}=\frac{K_{t}}{R}u-\frac{K_{t}K_{e}}{R}\dot{ο}-T_{s}-T_{f}\mathrm{sgn}(\omega )-B\omega -T_{L}.$$

Selecting the system state variables as $x={\left[\begin{array}{cc} ο & \dot{ο} \end{array}\right]}^{T}={\left[\begin{array}{cc} x_{1} & x_{2} \end{array}\right]}^{T}$. The EETS are formulated as follows:

(5) $$\left\{\begin{array}{l} {\dot{x}}_{1}=x_{2}\\ {\dot{x}}_{2}=h+gu+d \end{array}\right.$$

where $h\left(x_{1},x_{2}\right)=-\frac{K_{t}K_{e}}{RJ}x_{2},g=\frac{K_{t}}{RJ},d=-\frac{1}{J}\left(T_{s}+T_{f}\mathrm{sgn}(x_{2})+Bx_{2}\;+T_{L}\right)$.

**Remark** **1.** *The goal of control methodology is to derive a novel adaptive terminal sliding mode tracking control (ATSMC) strategy integrated with an enhanced disturbance observer for EETSs such that the EETS throttle angle precisely tracks the desired angle/foot pedal input as closely as possible.*

## 3. Controller Algorithm

This section presents the design of the proposed ESESO-based adaptive terminal sliding mode controller for the electronic throttle system. By integrating disturbance estimation and adaptive control mechanisms, the proposed method enhances tracking accuracy and robustness under composite disturbances. The schematic diagram of the control architecture is depicted in Figure 2, and the detailed implementation steps are given below:

### 3.1. Enhanced Super-Twisting Extended State Observer Design (ESESO)

**Lemma** **1**  ([33])**.** *For an arbitrary nonlinear system, there is a Lyapunov function V_m_* > 0 *and its derivative satisfy that* ${\dot{V}}_{m}\left(x\right)\;\leq -MV_{m}^{\upsilon }\left(x\right)$*,* M>0,0<υ<1*, then denote there exist a convergence finite-time that define as follows* $T_{f}\leq \frac{1}{M\left(1-\upsilon \right)}{\left|V_{m}\left(x\left(0\right)\right)\right|}^{1-\upsilon }$.

**Assumption** **1.** *The lumped disturbance d*(*t*)*, including external disturbances, parameter uncertainties, and unmodeled dynamics, is bounded and continuously differentiable. There exist unknown positive constants D and* χ *such that* ∣*d*(*t*)∣ ≤ *D and* ∣*d˙*(*t*)∣ ≤ χ *for all t* ≥ 0.

The ESESO can be established as follows:

(6) $$\left\{\begin{array}{l} {\dot{\hat{x}}}_{1}={\hat{x}}_{2}+\beta_{1}z_{1}\\ {\dot{\hat{x}}}_{2}=\hat{h}+gu+{\hat{x}}_{e}+f_{SW}\\ {\dot{\hat{x}}}_{e}=\lambda_{1}sign\left(\psi \right)+\lambda_{2}\psi \end{array}\right.$$

where ${\hat{x}}_{i},i=1,2,e$ denote the estimated value of the state $x_{i}$; $\beta_{1}$ denotes the constant observer gain; $\lambda_{i},i=1,2$ define the positive super-twisting switching constants, $f_{SW}$ defines the auxiliary term.

The estimation error of the ESESO can be computed as follows:

(7) $$\left\{\begin{array}{l} {\dot{z}}_{1}={\dot{x}}_{1}-{\dot{\hat{x}}}_{1}=z_{2}-\beta_{1}z_{1}\\ {\dot{z}}_{2}={\dot{x}}_{2}-{\dot{\hat{x}}}_{2}=z_{e}-f_{SW}+\tilde{h}\\ {\dot{z}}_{e}={\dot{x}}_{e}-{\dot{\hat{x}}}_{e}={\dot{x}}_{e}-\lambda_{1}sign\left(\psi \right)-\lambda_{2}\psi \end{array}\right.$$

where $z_{i}=x_{i}-{\hat{x}}_{i},i=1,2,e$ denote the state estimation errors.

The sliding manifold ψ for the ESESO can be selected as follows:

(8) $$\psi =z_{2}+\gamma z_{1}$$

where γ denotes the constant gain.

The auxiliary variable is defined as follows:

(9) $$f_{SW}=\gamma z_{2}-\beta_{1}\gamma z_{2}+\lambda_{11}{\left|\psi \right|}^{1/2}sign\left(\psi \right)+\lambda_{12}\psi +\tilde{h}$$

where $\lambda_{11}\;\mathrm{and}\;\lambda_{12}$ denote the constant gains, $\tilde{h}=h\left(x\right)-\hat{h}\left(\hat{x}\right)$.

Noting (9), the first and second derivative of ψ can be calculated as follows:

(10) $$\begin{array}{l} \dot{\psi }={\dot{z}}_{2}+\gamma {\dot{z}}_{1}=z_{e}-f_{SW}+\tilde{h}+\gamma \left(z_{2}-\beta_{1}z_{1}\right)\\ =z_{e}-\lambda_{11}{\left|\psi \right|}^{1/2}sign\left(\psi \right)-\lambda_{12}\psi \\ \ddot{\psi }={\dot{z}}_{e}-\frac{\lambda_{11}}{2{\left|\psi \right|}^{1/2}}\dot{\psi }-\lambda_{12}\dot{\psi } \end{array}$$

The Lyapunov function (LFU) is chosen as follows:

(11) $$V_{ESESO}=2\lambda_{1}\left|\psi \right|+\lambda_{2}\psi^{2}+\frac{1}{2}z_{e}^{2}+\frac{1}{2}{\dot{\psi }}^{2}$$

The time derivative of *V_ESESO_* is given as follows:

(12) $$\begin{array}{l} {\dot{V}}_{ESESO}=2\lambda_{1}sign\left(\psi \right)\dot{\psi }+2\lambda_{2}\psi \dot{\psi }+z_{e}{\dot{z}}_{e}+\dot{\psi }\ddot{\psi }\\ =2\lambda_{1}sign\left(\psi \right)\dot{\psi }+2\lambda_{2}\psi \dot{\psi }+z_{e}{\dot{z}}_{e}+\dot{\psi }\left({\dot{z}}_{e}-\frac{\lambda_{11}}{2{\left|\psi \right|}^{1/2}}\dot{\psi }-\lambda_{12}\dot{\psi }\right)\\ =-\frac{1}{{\left|\psi \right|}^{1/2}}\Phi^{T}P_{1}\Phi -\Phi^{T}Q_{1}\Phi +{\dot{x}}_{e}\left(\dot{\psi }+z_{e}\right)\\ \leq -\frac{1}{{\left|\psi \right|}^{1/2}}\Phi^{T}P_{1}\Phi -\Phi^{T}Q_{1}\Phi +\frac{1}{{\left|\psi \right|}^{1/2}}\Omega^{T}P_{2}\Omega +\Omega^{T}Q_{2}\Omega \end{array}$$

where

$$\begin{array}{l} \Phi ={\left[\begin{array}{ccc} {\left|\psi \right|}^{1/2}sign\left(\psi \right) & \psi & z_{e} \end{array}\right]}^{T};\Omega ={\left[\begin{array}{ccc} {\left|\psi \right|}^{1/2} & \left|\psi \right| & \left|z_{e}\right| \end{array}\right]}^{T},\left|\psi \right|\leq {\left\|\Omega \right\|}^{2}\\ P_{1}=\frac{\lambda_{11}}{2}\left[\begin{array}{ccc} 2\lambda_{1}+\lambda_{11}^{2} & 0 & -\lambda_{11}\\ 0 & 2\lambda_{2}-3\lambda_{12}^{2} & -3\lambda_{12}\\ -\lambda_{11} & -3\lambda_{12} & 1 \end{array}\right];Q_{1}=\lambda_{12}\left[\begin{array}{ccc} \lambda_{1} & 0 & 0\\ 0 & \lambda_{2}+\lambda_{12}^{2} & -\lambda_{12}\\ 0 & -\lambda_{12} & 1 \end{array}\right];\\ P_{2}=\left[\begin{array}{ccc} \chi \lambda_{11} & 0 & \chi \\ 0 & 0 & 0\\ \chi & 0 & 0 \end{array}\right];Q_{2}=\left[\begin{array}{ccc} \chi \lambda_{12} & 0 & 0\\ 0 & 0 & 0\\ 0 & 0 & 0 \end{array}\right];\left|{\dot{x}}_{e}\right|\leq \chi \end{array}$$

With properly designed gains $\lambda_{i}$(i=1, 2, 11, 12) satisfying $P_{1}-P_{2}>0,Q_{1}-Q_{2}>0$, it yields

(13) $${\dot{V}}_{ESESO}\leq -\frac{1}{{\left|\psi \right|}^{1/2}}\Omega^{T}\left(P_{1}-P_{2}\right)\Omega -\Omega^{T}\left(Q_{1}-Q_{2}\right)\Omega$$

It is noted that $V_{ESESO}$ can be expressed as

(14) $$V_{ESESO}=\Phi^{T}P\Phi ,P=\frac{1}{2}\left[\begin{array}{ccc} 4\lambda_{1}+\lambda_{11}^{2} & \lambda_{11}\lambda_{12} & -\lambda_{11}\\ \lambda_{11}\lambda_{12} & 2\lambda_{2}+\lambda_{12}^{2} & -\lambda_{12}\\ -\lambda_{11} & -\lambda_{12} & 2 \end{array}\right]$$

Following (13) and (14), it yields

(15) $$\begin{array}{l} \lambda_{\min}\left(P\right){\left\|\Omega \right\|}^{2}\leq V_{ESESO}\leq \lambda_{\max}\left(P\right){\left\|\Omega \right\|}^{2}\\ \to {\left\|\Omega \right\|}^{2}\geq \frac{V_{ESESO}}{\lambda_{\max}\left(P\right)},\left\|\Omega \right\|\geq \frac{V_{ESESO}^{1/2}}{\lambda_{\max}{\left(P\right)}^{1/2}} \end{array}$$

**Theorem** **1.** *For the EETS (5), under the ESESO designed in Equations (6) and (8) and the super-twisting law in Equation (9), the disturbance estimation errors* $z_{i},i=1,2,e$ *are bounded and convergence to the zero in the finite time T_f_.*

**Proof** **of** **Theorem** **1.** The ${\dot{V}}_{ESESO}$ can be re-expressed by

(16) $$\begin{array}{l} {\dot{V}}_{ESESO}\leq -\frac{1}{{\left|\psi \right|}^{1/2}}\lambda_{\min}\left(P_{1}-P_{2}\right){\left\|\Omega \right\|}^{2}-\lambda_{\min}\left(Q_{1}-Q_{2}\right){\left\|\Omega \right\|}^{2}\\ \leq -\frac{\lambda_{\min}\left(P_{1}-P_{2}\right)}{\lambda_{\max}{\left(P\right)}^{1/2}}V_{ESESO}^{1/2}-\frac{\lambda_{\min}\left(Q_{1}-Q_{2}\right)}{\lambda_{\max}\left(P\right)}V_{ESESO}\\ \leq -\frac{\lambda_{\min}\left(P_{1}-P_{2}\right)}{\lambda_{\max}{\left(P\right)}^{1/2}}V_{ESESO}^{1/2} \end{array}$$

Based on Lemma 1, the sliding manifold ψ and the error $z_{e}$ converge to zero within a finite time *T_f_*, where $T_{f}\leq \frac{2\lambda_{\max}{\left(P\right)}^{1/2}}{\lambda_{\min}\left(P_{1}-P_{2}\right)}V_{ESESO}{\left(0\right)}^{1/2}$.

Therefore, Theorem 1 is demonstrated. □

**Remark** **2.** *The gain tuning procedure of the ESESO are selected as the following. First, a positive* $\lambda_{11}$ *and* $\lambda_{12}$ *according to the desired observer convergence speed under the specified bound* χ. *Next,* $\lambda_{2}$ *is selected sufficiently large to satisfy* $\lambda_{2}>6\lambda_{12}^{2}\;$ *and* $\lambda_{1}$ *is selected sufficiently large to satisfy the corresponding lower bound. Then,* $P_{1}-P_{2}$ *and* $Q_{1}-Q_{2}$ *are constructed using the selected gains and their eigenvalues are calculated. The gains are increased if necessary until* $\min\left\{\mathrm{eig}\left(P_{1}-P_{2}\right)\right\}>0$ *and* $min\left\{eig\left(Q_{1}-Q_{2}\right)\right\}>0.$ *Finally, the practical values of these gains are fine-tuned through trial-and-error experimental tests to ensure that the estimated states closely track the corresponding measured states.*

### 3.2. Synthesis Controller Design

The tracking error is expressed as follows:

(17) $$\begin{array}{l} e_{1}={\hat{x}}_{1}-x_{1d},\\ e_{2}={\hat{x}}_{2}-{\dot{x}}_{1d}, \end{array}$$

where *x*_1*d*_ defines the reference signal.

Differentiating (17) with respect to time, yields:

(18) $$\begin{array}{l} {\dot{e}}_{1}=e_{2},\\ {\dot{e}}_{2}={\dot{\hat{x}}}_{2}-{\ddot{x}}_{1d}=\hat{h}+gu+{\hat{x}}_{e}+f_{SW}-{\ddot{x}}_{1d}, \end{array}$$

The terminal sliding manifold is introduced as follows:

(19) $$\begin{array}{l} \sigma ={\dot{e}}_{2}+\eta_{1}sign\left(e_{1}\right){\left|e_{1}\right|}^{\rho_{1}}+\eta_{2}sign\left(e_{2}\right){\left|e_{2}\right|}^{\rho_{2}}\\ =h+gu+{\hat{x}}_{e}+f_{SW}-{\ddot{x}}_{1d}+\eta_{1}sign\left(e_{1}\right){\left|e_{1}\right|}^{\rho_{1}}+\eta_{2}sign\left(e_{2}\right){\left|e_{2}\right|}^{\rho_{2}}, \end{array}$$

where $\rho_{1}=\frac{\rho_{2}\rho_{3}}{2\rho_{3}-\rho_{2}},\rho_{2}=\rho ,\rho_{3}=1$, 0<ρ<1 defines a designed positive scalar. $\eta_{1}\;\mathrm{and}\;\eta_{2}$ are chosen to ensure that the resulting characteristic polynomial $s^{2}+\eta_{2}s+\eta_{1}$ is a Hurwitz polynomial, *s* denote Laplace operator.

The equivalent control law is introduced as follows:

(20) $$u_{q}=\frac{1}{g}\left({\ddot{x}}_{1d}-\hat{h}-f_{SW}-\eta_{1}sign\left(e_{1}\right){\left|e_{1}\right|}^{\rho_{1}}-\eta_{2}sign\left(e_{2}\right){\left|e_{2}\right|}^{\rho_{2}}-{\hat{x}}_{e}\right)$$

The robust control law is introduced as follows:

(21) $$\begin{array}{l} {\dot{u}}_{r}+\tau u_{r}=u_{0}\\ u_{0}=\frac{1}{g}\left(-{\hat{\eta }}_{3}sign\left(\sigma \right)-\eta_{4}\sigma \right) \end{array}$$

where ${\hat{\eta }}_{3}\;\mathrm{and}\;\eta_{4}$ define the designed adoption gain and positive scalar, τ>0.

The adaptive law is developed as follows:

(22) $${\hat{\eta }}_{3}=\kappa_{0}\int_{0}^{t}\exp\left(\kappa_{1}\left|\sigma \right|\right)\left|\sigma \right|dt$$

where $\kappa_{0}\;\mathrm{and}\;\kappa_{1}$ define the positive scalars, ${\hat{\eta }}_{3}\leq \eta_{0},\eta_{0}>0$.

The final control law is designed as follows:

(23) $$u=u_{r}+u_{q}$$

According to (19) and (21), one obtains:

(24) $$\begin{array}{l} \sigma =gu_{r}+\tilde{h}\\ \dot{\sigma }=g{\dot{u}}_{r}+\dot{\tilde{h}}=g\left(u_{0}-\tau u_{r}\right)+\dot{\tilde{h}}\\ =-{\hat{\eta }}_{3}sign\left(\sigma \right)-\eta_{4}\sigma -g\tau u_{r}+\dot{\tilde{h}} \end{array}$$

The LFU is designed as follows:

(25) $$V=\frac{1}{2}\sigma^{2}+\frac{1}{2}{\tilde{\eta }}_{3}^{2}$$

where ${\tilde{\eta }}_{3}={\hat{\eta }}_{3}-\eta_{0},\left|{\tilde{\eta }}_{3}\right|\leq \eta_{b}$.

Taking derivative of (25), yields:

(26) $$\begin{array}{l} \dot{V}=\sigma \dot{\sigma }+{\tilde{\eta }}_{3}{\dot{\hat{\eta }}}_{3}\\ =\sigma \left(-{\hat{\eta }}_{3}sign\left(\sigma \right)-\eta_{4}\sigma -g\tau u_{r}+\dot{\tilde{h}}\right)+\left({\hat{\eta }}_{3}-\eta_{0}\right)\kappa_{0}\exp\left(\kappa_{1}\left|\sigma \right|\right)\left|\sigma \right|\\ =-\eta_{0}\left|\sigma \right|-\eta_{4}\sigma^{2}-\sigma g\tau u_{r}+\sigma \dot{\tilde{h}}+\left({\hat{\eta }}_{3}-\eta_{0}\right)\left(\kappa_{0}\exp\left(\kappa_{1}\left|\sigma \right|\right)-1\right)\left|\sigma \right|\\ \leq -\eta_{0}\left|\sigma \right|-\eta_{4}\sigma^{2}-\sigma g\tau u_{r}+\sigma \dot{\tilde{h}}-\eta_{a}\left|{\tilde{\eta }}_{3}\right| \end{array}$$

where $\eta_{a}=\left(\kappa_{0}\exp\left(\kappa_{1}\left|\sigma \right|\right)\left|\sigma \right|-1\right)\left|\sigma \right|$.

Based on Theorem 1 and applying Young’s inequality, one gets:

(27) $$\begin{array}{l} -\sigma g\tau u_{r}\leq \frac{1}{2}\sigma^{2}+\frac{\tau \left|g\right|}{2}u_{m}^{2}\\ \sigma \dot{\tilde{h}}\leq \frac{1}{2}\sigma^{2}+\frac{1}{2}h_{m}^{2} \end{array}$$

where $u_{m}\;\mathrm{and}\;h_{m}$ represent the upper bound of $u_{r}\;\mathrm{and}\;\dot{\tilde{h}}$.

By substituting (27) into (26), one yields:

(28) $$\begin{array}{l} \dot{V}\leq -\eta_{0}\left|\sigma \right|-\left(\eta_{4}-1\right)\sigma^{2}-\eta_{a}\left|{\tilde{\eta }}_{3}\right|-\eta_{a}{\tilde{\eta }}_{3}^{2}+\frac{\tau \left|g\right|}{2}u_{m}^{2}+\frac{1}{2}h_{m}^{2}+\eta_{a}\eta_{b}^{2}\\ \dot{V}\leq -N_{1}V-N_{2}V^{\frac{1}{2}}+T \end{array}$$

where $N_{1}=\;\lambda_{min}\left(\eta_{3}\right)\;,N_{2}=\;\lambda_{min}\left(\eta_{4}-1\right)\;,\;\mathrm{and}\;T=\frac{\tau \left|g\right|}{2}u_{m}^{2}+\frac{1}{2}h_{m}^{2}+\eta_{a}\eta_{b}^{2}$.

**Theorem** **2.**  *For the EETS (5), under the proposed ESESO (6), the proposed sliding manifold (19), the equivalent and robust control law (20), (21), the error tracking variables* $e_{i}$ *in (17) and other states of the EETS satisfies that* $e_{i},\sigma \to 0\;\mathrm{as}\;t\to \infty ,i=1,2$ *in the finite-time.*

**Proof** **of** **Theorem** **2.** According to (28), the region of solution of EETS is described by

(29) $$\underset{t\to T_{r}}{\lim}V(x)\leq \min\left\{\frac{T}{\left(1-\varepsilon_{0}\right)N_{1}},{\left(\frac{T}{\left(1-\varepsilon_{0}\right)N_{2}}\right)}^{2}\right\}$$

where $\varepsilon_{0}\;\mathrm{satisfies}\;0<\varepsilon_{0}<1$.

It can be revealed that *V* is bounded; the time of convergence is given by [34,35].

(30) $$T_{r}\leq \max\left\{t_{0}+\frac{2}{\varepsilon_{0}N_{1}}\ln\frac{\varepsilon_{0}N_{1}V^{\frac{1}{2}}\left(t_{0}\right)+N_{2}}{N_{2}}\right.\left.t_{0}+\frac{2}{N_{1}}\ln\frac{N_{1}V^{\frac{1}{2}}\left(t_{0}\right)+\varepsilon_{0}N_{2}}{\varepsilon_{0}N_{2}}\right\}$$

Hence, Theorem 2 is completed. □

**Remark** **3.** *To mitigate the chattering phenomenon, the discontinuous sign function in (19)–(21) is replaced by a saturation function as follows:*

(31) $$sat\left(\frac{\sigma }{\varepsilon_{\sigma }}\right)=\left\{\begin{array}{l} sign\left(\sigma \right)\;\mathrm{if}\;\left|\sigma \right|\geq \varepsilon_{\sigma }\\ \frac{\sigma }{\varepsilon_{\sigma }}\;\mathrm{otherwise} \end{array}\right.$$

*where ε_σ_ is a small positive coefficient*.

**Remark** **4.** *For parameter selection guidelines, the parameter ρ determines the nonlinear characteristics and convergence behavior of the terminal sliding manifold. A smaller value of ρ generally enhances the terminal nonlinear effect, whereas a value closer to one results in a smoother response. Therefore, ρ is selected by considering the trade-off between convergence speed, control smoothness, and measurement-noise sensitivity. Meanwhile, the parameters η*_1_ *and η*_2_ *are specified from a desired natural frequency ω_n_ and damping ratio ζ according to* $\eta_{1}=\omega_{n}^{2},\eta_{2}=2\omega_{n}\zeta$ *such that is Hurwitz. A larger ω_n_ provides faster convergence but may increase control activity and sensitivity to measurement noise, while ζ can be adjusted to regulate the transient damping. For* $\kappa_{0}$ *and* $\kappa_{1}$ *, appropriately increasing their values can accelerate error convergence. In contrast,* $\eta_{4}$ *should be carefully tuned to achieve a balance between tracking performance and physical constraints.*

## 4. Results and Discussion

### 4.1. Experimental Setup

To verify the superiority of the novel method, comparative experiments were carried out in the MATLAB/Simulink ver. 2024b environment using two representative reference trajectories. The first trajectory is a pulse signal with an amplitude of 45°, a period of 5 s, and a duty cycle of 50%. The second trajectory is a sinusoidal signal given by *x*_1*d*_ = 30 + 30sin(t/p) (degree), where t ∈ [0, 20].

The parameters of the above-mentioned EETS are listed in Table 1 as follows:

Three other control strategies are considered for performance comparison: TSMC controller without adaptive law and ESESO (S1), terminal sliding mode controller with adaptive law without ESESO (S2), and adaptive sliding mode controller without adaptive law with ESESO (S3).

The gain parameters of the proposed methodology are designed according to Remarks 2 and 4 as follows:

$$\begin{array}{l} ESESO:\beta_{1}=15,\lambda_{1}=100,\lambda_{2}=23,\gamma =1.6,\lambda_{11}=9,\lambda_{12}=1,\\ ATSMC:\rho =0.6,\eta_{1}=25,\eta_{2}=10,\eta_{4}=7,\kappa_{0}=0.18,\kappa_{1}=0.7 \end{array}$$

**Remark** **5.** *To ensure a fair comparison, the gains of the three benchmark controllers, namely S1 (TSMC), S2 (ATSMC), and S3 (TSMC-ESO), were kept the same across the benchmark controllers. This was intentionally done to provide a consistent basis for comparison and to avoid giving any benchmark controller an advantage through controller-specific gain tuning.*

Figure 3 presents the hardware implementation of the EETS (Peugeot 3008 model) experimental platform used for controller validation. The system consists of a personal computer (PC), microcontroller, motor driver, automotive battery, throttle valve assembly, angle sensor, foot pedal, and a circuit breaker for protection. The component setting devices of the practical EETS are shown in Table 2. The control loop is executed with a sampling time of 0.01 s. The PC serves as the host computer for controller implementation in MATLAB/SIMULINK environment, data acquisition, and real-time monitoring. The microcontroller executes the control algorithm and communicates with the PC. The motor driver (SmartDriveDuo-30) amplifies the control signal generated by the microcontroller to actuate the DC motor integrated within the throttle valve. An automotive battery provides the required power supply for the actuator and electronic components, while the circuit breaker ensures safe operation against overcurrent conditions. The throttle valve is equipped with an angle sensor that measures the throttle plate position and provides feedback for closed-loop control. The foot pedal acts as the driver input, generating the desired throttle opening command. The measured throttle angle is transmitted to the microcontroller, where it is compared with the reference signal to generate the appropriate control action.

This experimental setup enables real-time evaluation of the proposed control strategy under practical operating conditions and facilitates the assessment of the tracking accuracy, disturbance rejection capability, and control performance of the electronic throttle valve system.

### 4.2. Experimental Results

Case 1: The experimental results in this case are depicted in Figure 4, Figure 5, Figure 6 and Figure 7.

Figure 4 presents the tracking responses of the four controllers under a pulse reference signal that has a significant change from 0° to 45° at t = 0 and 5 s. All controllers are capable of following the reference trajectory; however, their tracking quality differs significantly. The (S1) exhibits noticeable oscillations around the reference value throughout the experiment due to limited disturbance rejection capability and the adaptive law. Compared with S1, the oscillation amplitude of S2 is reduced; nevertheless, some tracking deviations are still observed, particularly under high-frequency disturbances. The (S3) demonstrates stronger robustness against uncertainties and disturbances. The output remains concentrated around the reference trajectory with smaller tracking deviations than S1 and S2. Among all controllers, S4 provides the smoothest tracking response and the smallest visible deviation from the desired signal thanks to the assistance of ESESO and the adaptive gain law.

Figure 5 illustrates the tracking errors associated with each control strategy. The S1 controller exhibits the largest error amplitude, with oscillations extending over a wide range throughout the simulation. The S2 controller achieves a noticeable reduction in error magnitude compared with S1. The S3 controller maintains a narrower error band around zero than both S1 and S2. S4 produces the smallest error fluctuations among all methods. The error remains concentrated near zero during both steady-state operation and after the reference transition, confirming its effectiveness in maintaining accurate position tracking.

Figure 6 compares the control efforts generated by the four controllers. The S4 controller produces the smoothest control signal among all four methods. The control signal remains within a relatively narrow operating range while still achieving excellent tracking accuracy. Figure 7 shows the disturbance estimation results obtained by the proposed ESESO under pulse reference tracking scenario. As can be seen in Figure 7, the estimated disturbance exhibits rapid transitions corresponding to the abrupt changes in the reference signal. The observer accurately captures the disturbance variations during both rising and falling edges, demonstrating a fast convergence rate and strong transient estimation capability. This indicates that the ESESO effectively reconstructs the lumped disturbance while maintaining robustness against measurement noise and abrupt operating condition changes.

Case 2: The experimental results in this case are depicted in Figure 8, Figure 9, Figure 10 and Figure 11.

Figure 8 compares the trajectory tracking performance of the S1 to S4 controllers against the desired angle trajectory. All controllers are capable of following the reference signal; however, noticeable differences can be observed in their tracking accuracy. The S1 controller exhibits larger deviations from the reference trajectory, particularly during the transient periods where the reference changes rapidly. The S2 controller achieves better robustness against uncertainties and disturbances, allowing the system output to remain closer to the desired trajectory over most of the operating range. The S3 controller improves the tracking response due to the adaptive compensation capability. The S4 controller provides the closest agreement with the reference signal, demonstrating superior tracking accuracy and smoother response during both rising and falling segments of the trajectory. Although measurement noise is visible in all responses, the S4 controllers maintain smaller deviations from the desired angle.

The tracking error responses are presented in Figure 9. The error dynamics provide a clearer comparison of controller performance. The S1 controller exhibits relatively large oscillatory errors, especially during the initial transient stage and around regions of rapid trajectory variation. The S2 controller effectively reduces the error magnitude; however, some fluctuations remain due to the influence of disturbances and nonlinear dynamics. The S3 controller achieves low tracking errors and exhibits smoother error evolution. The S4 controller generally maintains the smallest error magnitude throughout most of the simulation period, demonstrating strong disturbance rejection and robustness. The error remains bounded within a narrow range around zero, indicating accurate tracking performance.

Figure 10 illustrates the control efforts generated by the four controllers. The actual control input applied to the EETS actuator is within [−10, 10] V, consistent with the voltage range of the experimental actuator and the practical constraints of the experimental setup. As seen, the S1 controller offers smooth but less accurate control, S2 improves adaptability, S3 achieves the best robustness at the expense of increased control activity, and S4 provides a good compromise between tracking performance and control smoothness. Figure 11 shows the disturbance estimation results obtained by the proposed ESESO under sinusoidal reference tracking scenario. The accurate disturbance estimation provided by the ESESO enables effective feedforward compensation within the control loop. By compensating for the estimated lumped disturbance in real time, the controller significantly reduces the influence of uncertainties and external perturbations on the tracking performance.

### 4.3. Performance Comparisons

#### 4.3.1. Tracking Error

For a clear verification, the above-mentioned experimental studies of the four controllers are tabulated in Table 3. The error indices, namely, mean absolute error (ME) and root mean square error (RSE), are described as follows:

In Case 1, the conventional TSMC (S1) exhibits the largest tracking error, with an RSE of 8.339 and an ME of 8.2095. By introducing the adaptive mechanism, ATSMC (S2) reduces the RSE and ME to 6.4381 and 6.3177, corresponding to improvements of approximately 22.8% and 23.0%, respectively. The incorporation of the ESO further enhances the disturbance rejection capability, enabling TSMC-ESO (S3) to achieve an RSE of 6.3716 and an ME of 6.1008. The proposed ATSMC-ESESO (S4) achieves the best performance, reducing the RSE and ME to 4.1742 and 4.1025, respectively. Compared with the conventional TSMC, the proposed method decreases the RSE by 49.9% and the ME by 50.0%, demonstrating the effectiveness of combining adaptive terminal sliding mode control with the enhanced disturbance observer.

In Case 2, all controllers exhibit improved tracking performance due to the smoother reference trajectory. Nevertheless, significant differences remain among the compared methods. TSMC produces an RSE of 4.3227 and an ME of 3.8826, while ATSMC reduces these values to 3.5808 and 3.0415, respectively. The introduction of the ESO dramatically improves the tracking accuracy, yielding an RSE of 1.1593 and an ME of 0.9125. The proposed ATSMC-ESESO achieves the smallest errors, with an RSE of only 0.4735 and an ME of 0.4366. Relative to TSMC, the proposed method reduces the RSE by approximately 89.0% and the ME by 88.8%.

#### 4.3.2. Chattering Phenomena

To quantitatively evaluate the chattering attenuation, several measures are calculated from the control input *u*, rather than relying solely on visual inspection of the control-signal response. Two useful quantitative chattering indices are total variation (TV) and root mean square of the control input (RMSU).

1. For sampled control input u(k), define the total variation (TV):

(32) $$TV\left(u\right)=\sum_{k=1}^{N-1}\left|u\left(k-1\right)-u\left(k\right)\right|$$

2. Root Mean Square of $\dot{u}$ (RMSU):
  (33) $$RMSU=\sqrt{\frac{1}{N-1}\sum_{k=1}^{N-1}{\left(\frac{u\left(k-1\right)-u\left(k\right)}{T_{s}}\right)}^{2}}$$
  where *T_s_* is the sampling time.

From Table 4, it can be seen that the proposed method (ATSMC-ESESO) has the smallest TV and RMSU for both indices, demonstrating that the integration of the ESESO with the ATSMC framework not only improves tracking accuracy but also effectively alleviates chattering in the control input.

To further evaluate the practical tracking capability of the proposed S4 (ATSMC + ESESO), an additional experiment is conducted using a randomly varying reference trajectory derived from the pedal-foot input. The experimental results are presented in Figure 12. As shown in Figure 12a, the actual steering angle closely follows the reference trajectory throughout the entire 25 s test, despite the rapid and irregular variations in the desired angle. The error in Figure 12b remains concentrated around zero for most of the experiment, although several transient deviations occur when the reference signal changes rapidly. The RMSE is 1.37°, and the maximum positive error is approximately 6°, while the negative error is generally within approximately −3.5°. The corresponding control input is shown in Figure 12c within approximately −1 to 8 V. These results further demonstrate that the proposed S4 (ATSMC + ESESO) is capable of accurately tracking realistic pedal-foot reference trajectories while maintaining bounded tracking errors and a smooth control effort.

The obtained performance comparisons demonstrate the effectiveness and practicality of the proposed control framework for high-performance electronic throttle applications under complex operating conditions. Furthermore, the enhanced observer provided highly accurate disturbance estimation, enabling efficient disturbance rejection without significantly increasing control effort.

## 5. Conclusions

This article presented a novel adaptive TSMC scheme for an EETS operating under composite disturbances and model uncertainties. By incorporating the ESESO output into the controller design, the impact of disturbances on the tracking control performance was effectively mitigated. An ATSMC was subsequently constructed to achieve finite-time convergence of the tracking error while eliminating the requirement for prior information of disturbance bounds. The adaptive mechanism automatically adjusted the control gain according to the EETS operating conditions, thereby reducing control conservatism and alleviating chattering effects. Rigorous Lyapunov-based analysis demonstrated the stability of the closed-loop system and ensured the finite-time convergence of errors. The experimental results on a real-time electronic throttle valve test bench showed that the proposed approach achieved superior tracking accuracy, faster transient response, and stronger robustness against composite disturbances compared with conventional control methods.

Future work will extend the proposed method to fault-tolerant control scenarios, networked automotive control systems, and intelligent vehicles operating under more complex driving conditions.

## Figures and Tables

**Figure 1:**
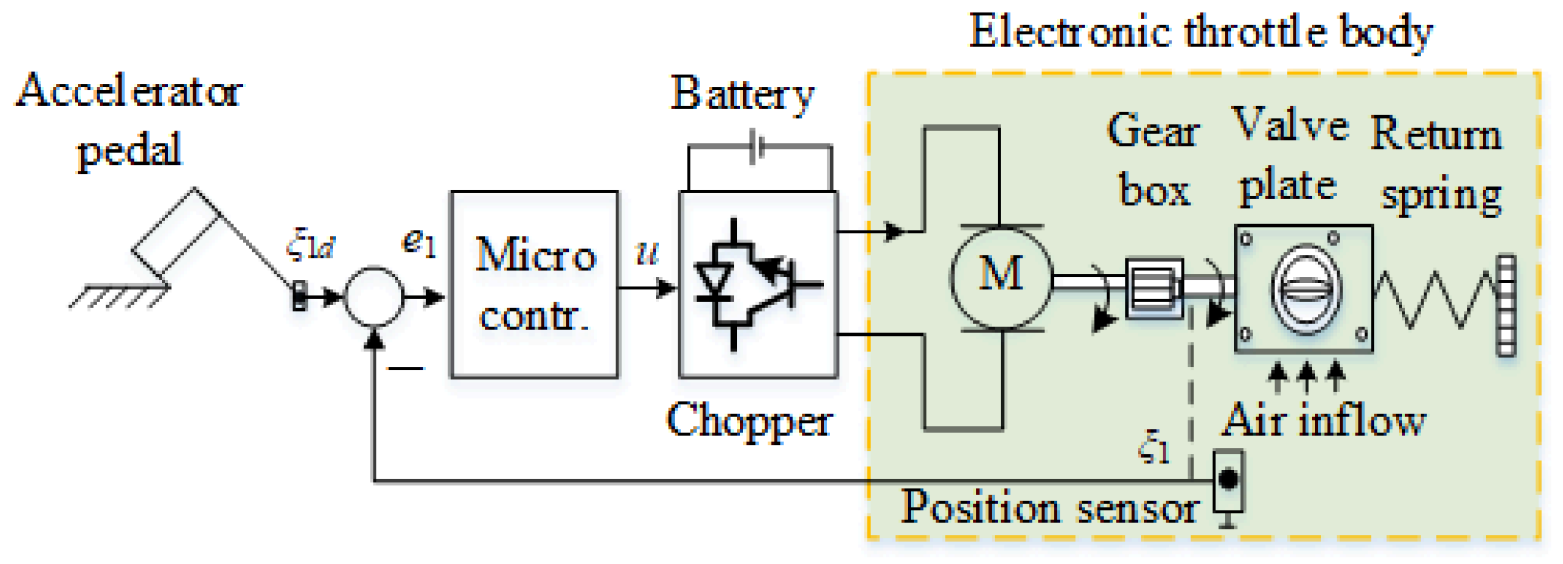
Configuration of the EETS.

**Figure 2:**
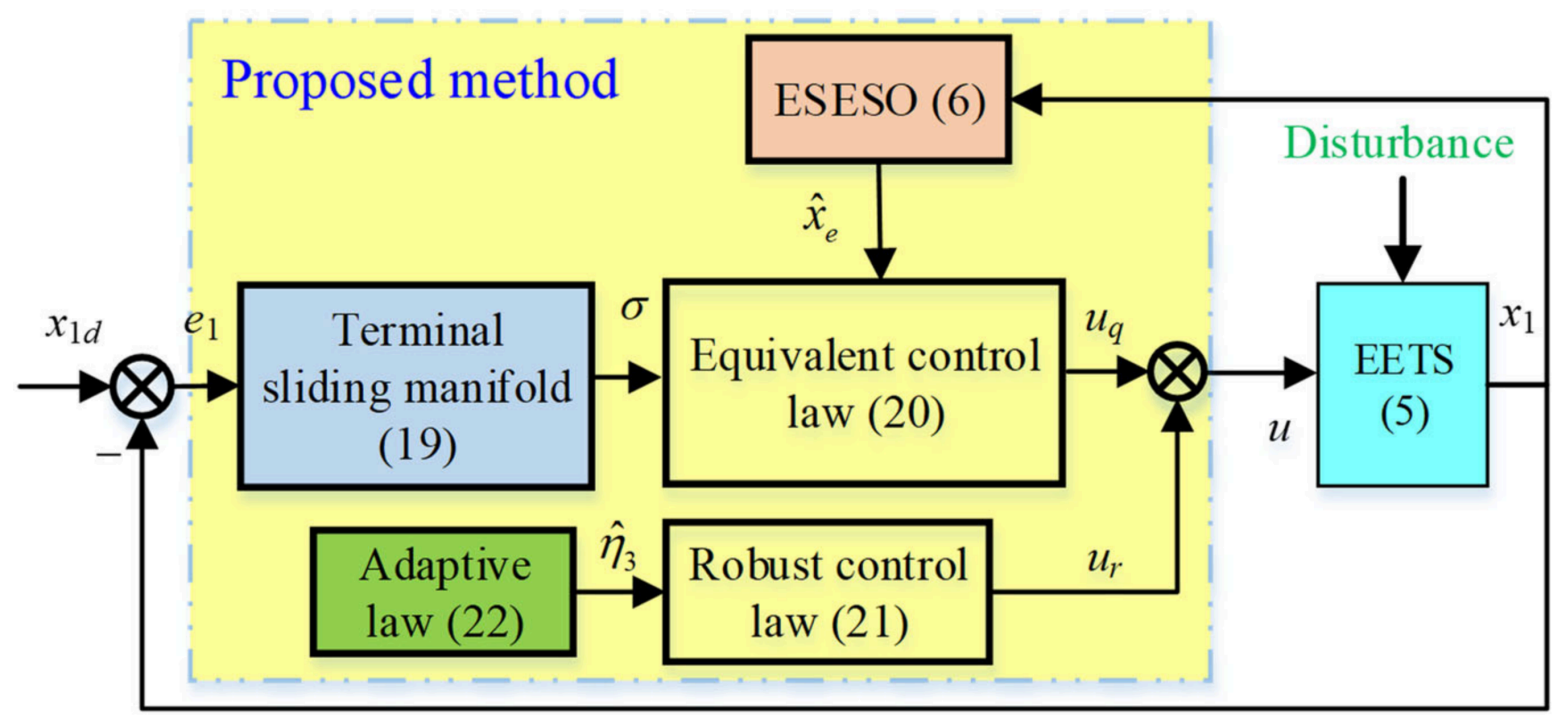
Topology of the proposed method.

**Figure 3:**
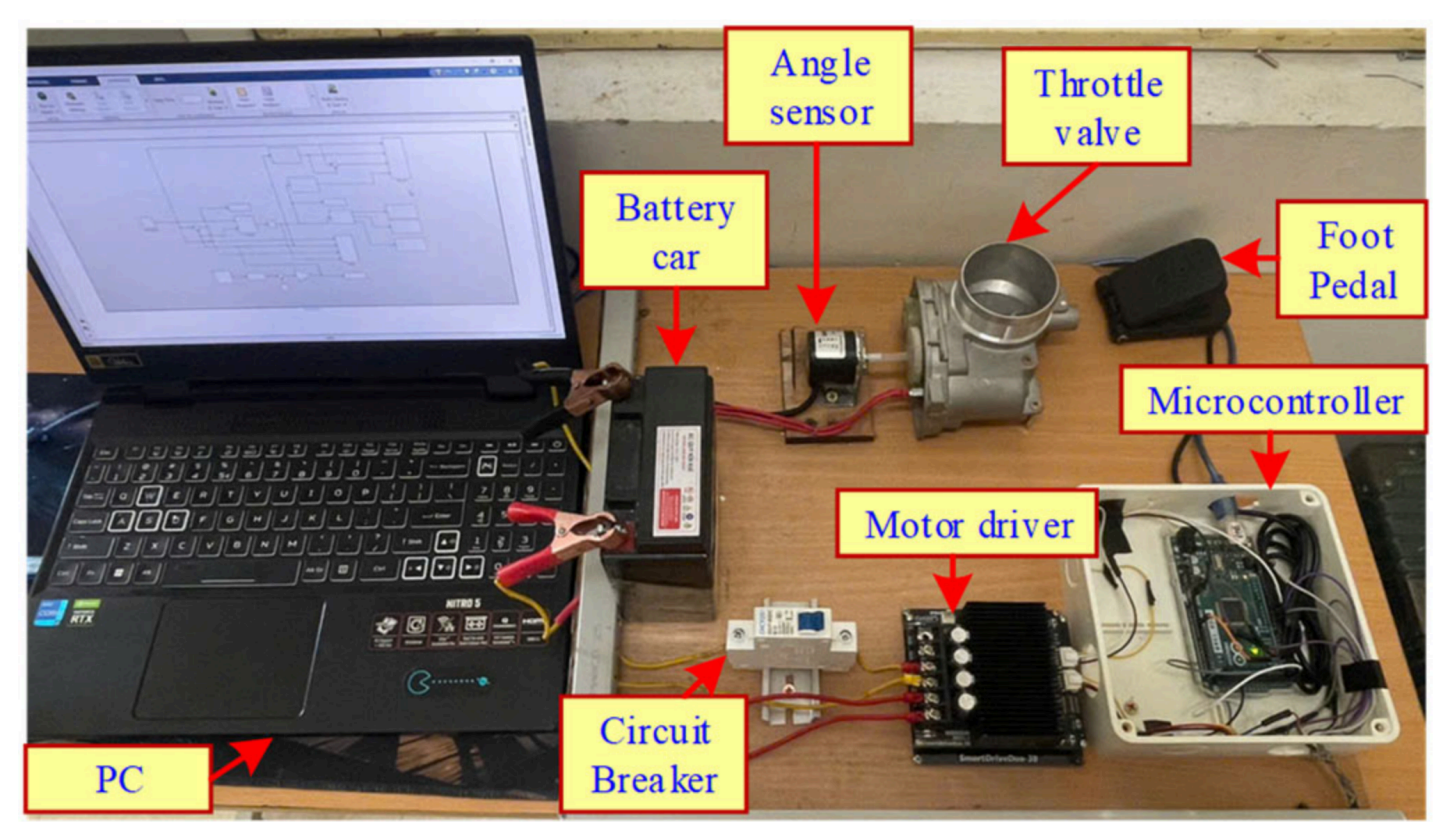
Experimental setup of the EETS test bench.

**Figure 4:**
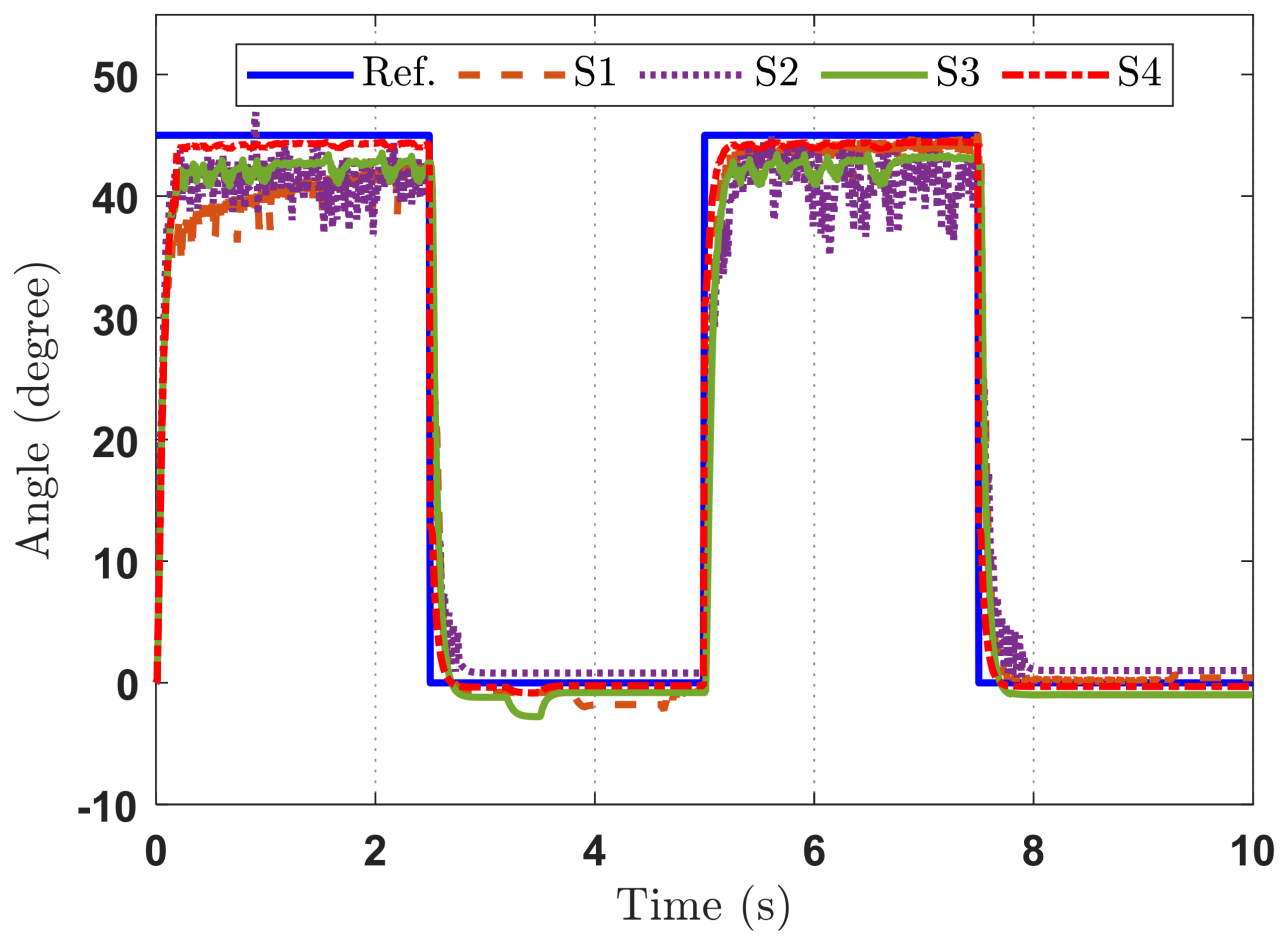
Throttle angle tracking curve under Case 1.

**Figure 5:**
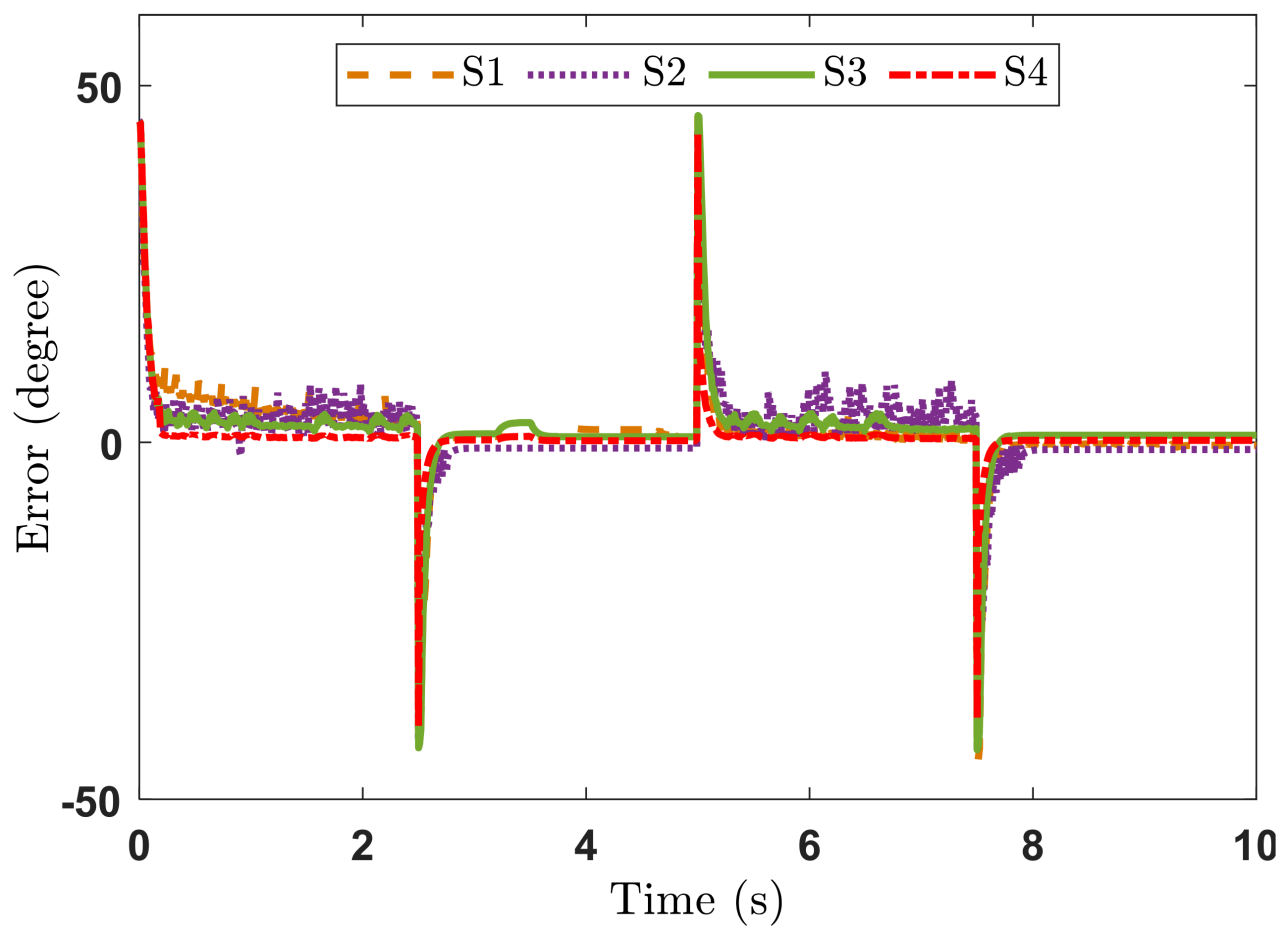
Curves of the angle tracking error under Case 1.

**Figure 6:**
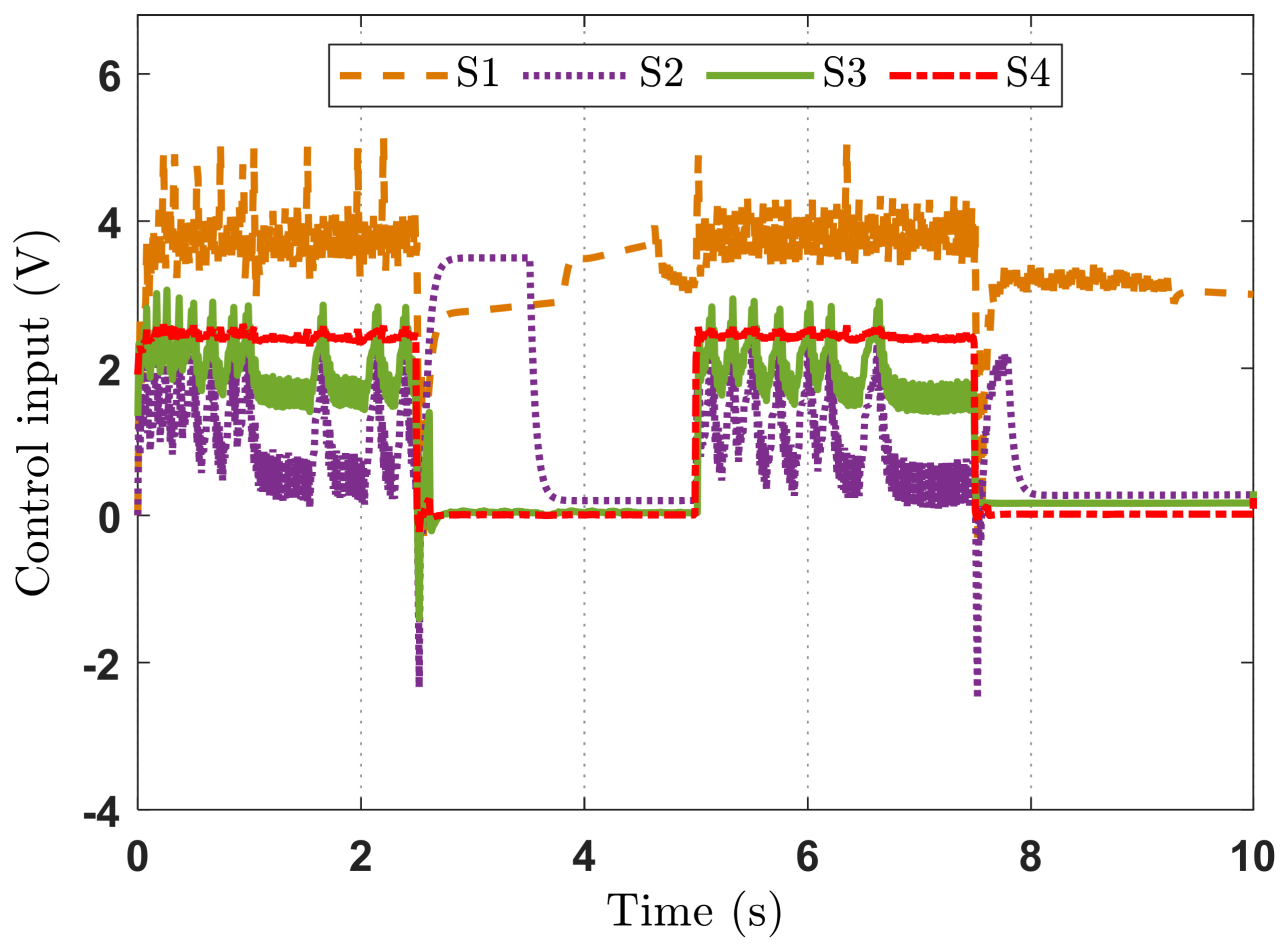
Responses of control inputs under Case 1.

**Figure 7:**
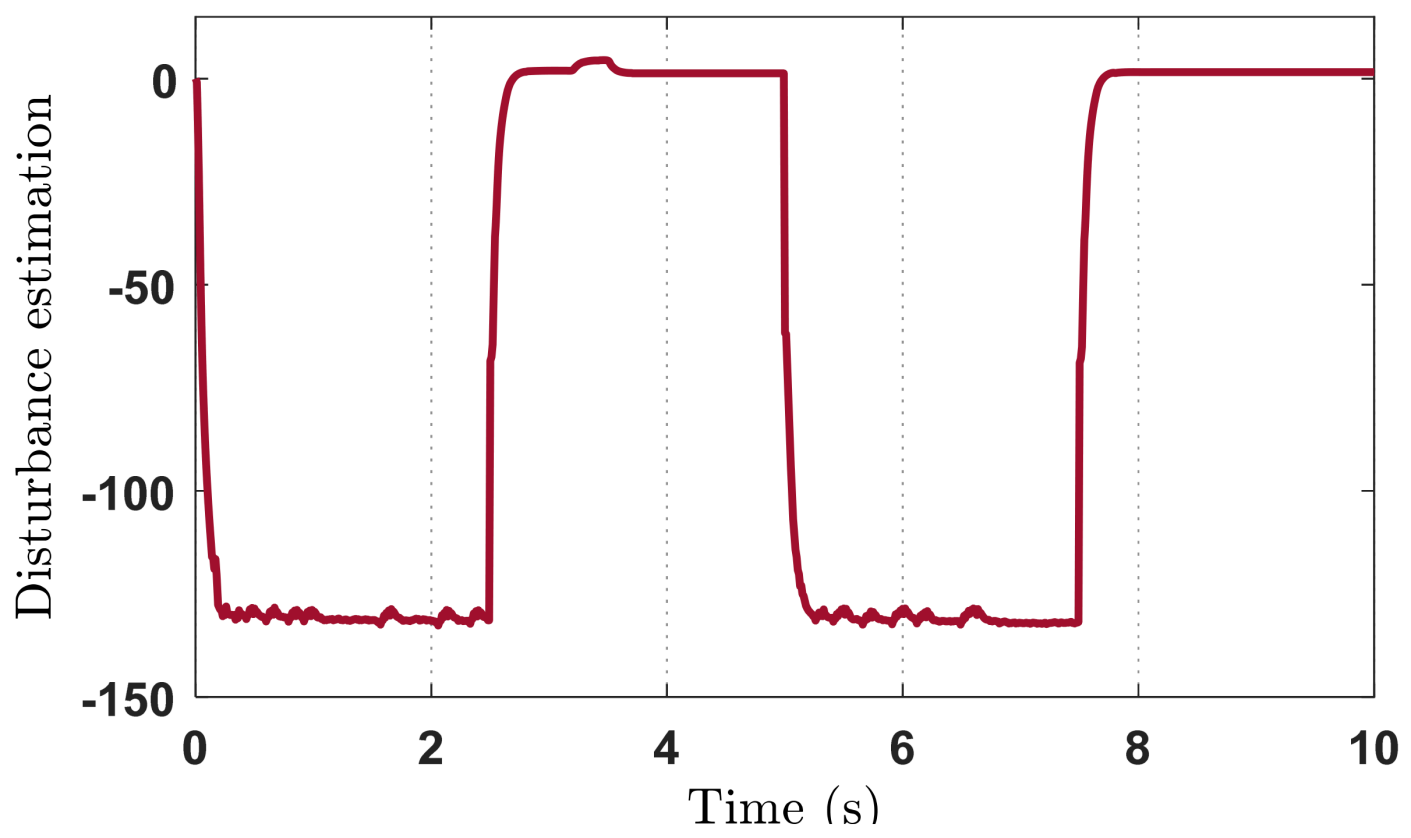
The estimated disturbances of the ESESO under Case 1.

**Figure 8:**
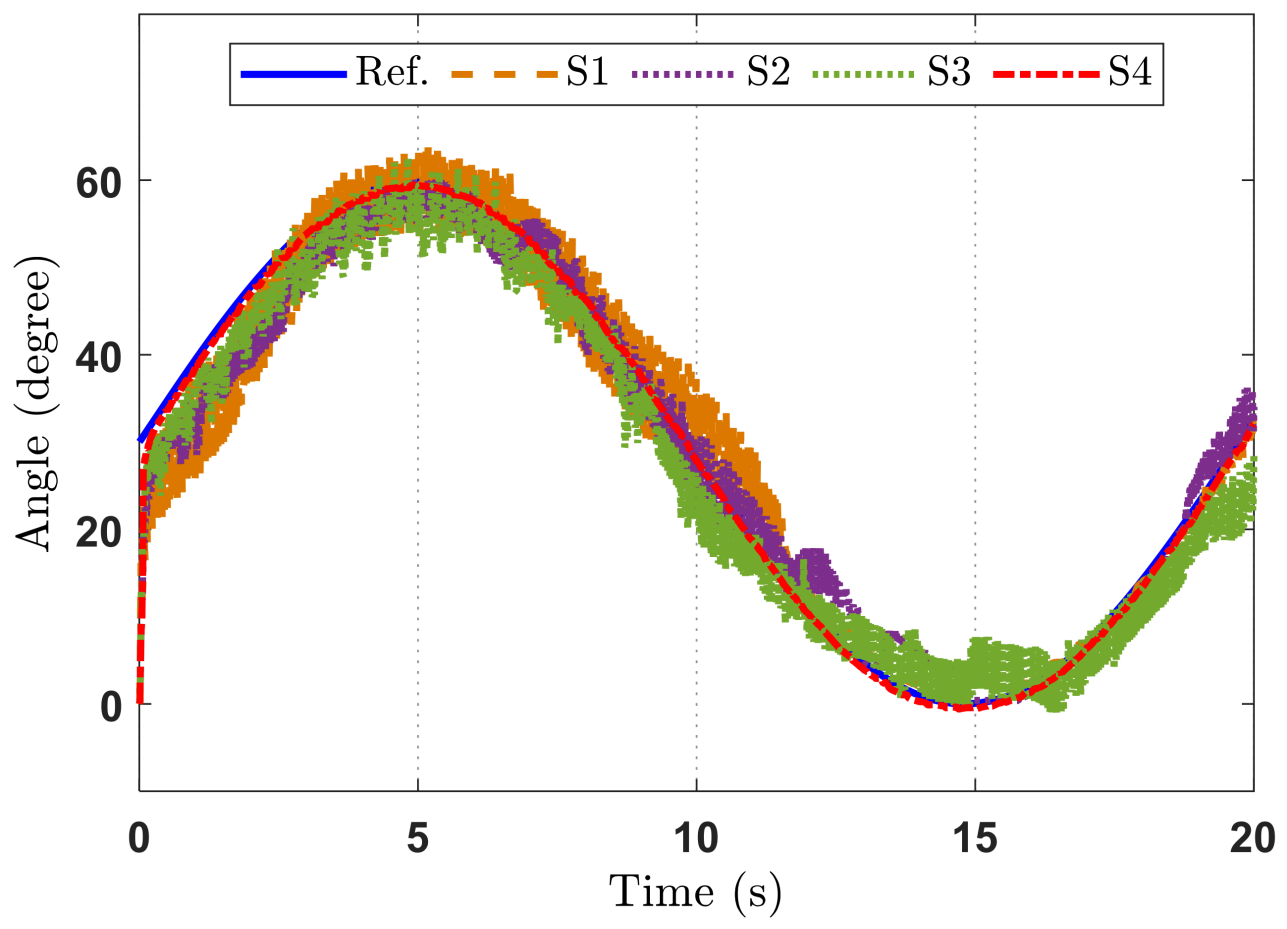
Throttle angle tracking curve under Case 2.

**Figure 9:**
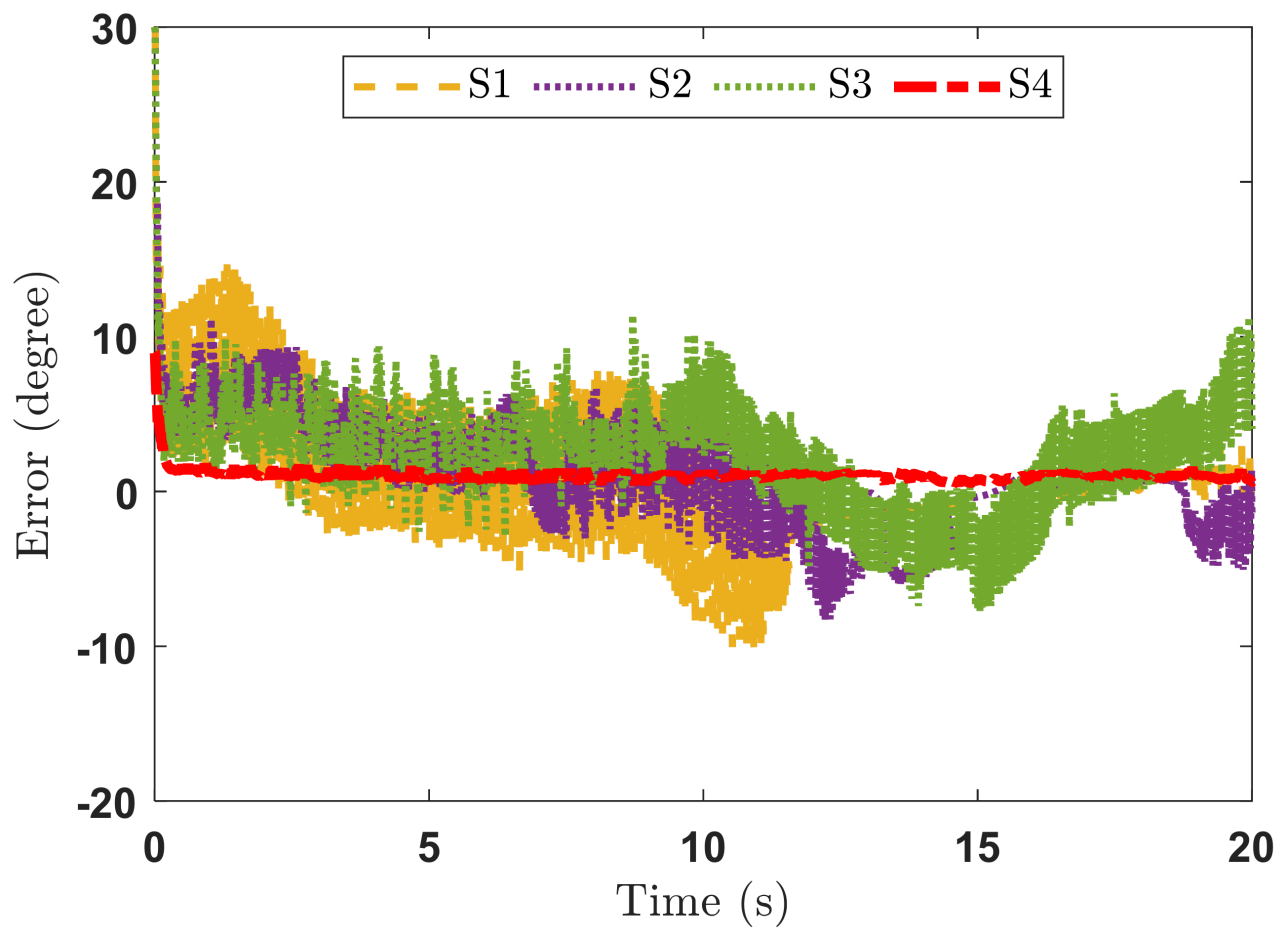
Curves of the angle tracking error under Case 2.

**Figure 10:**
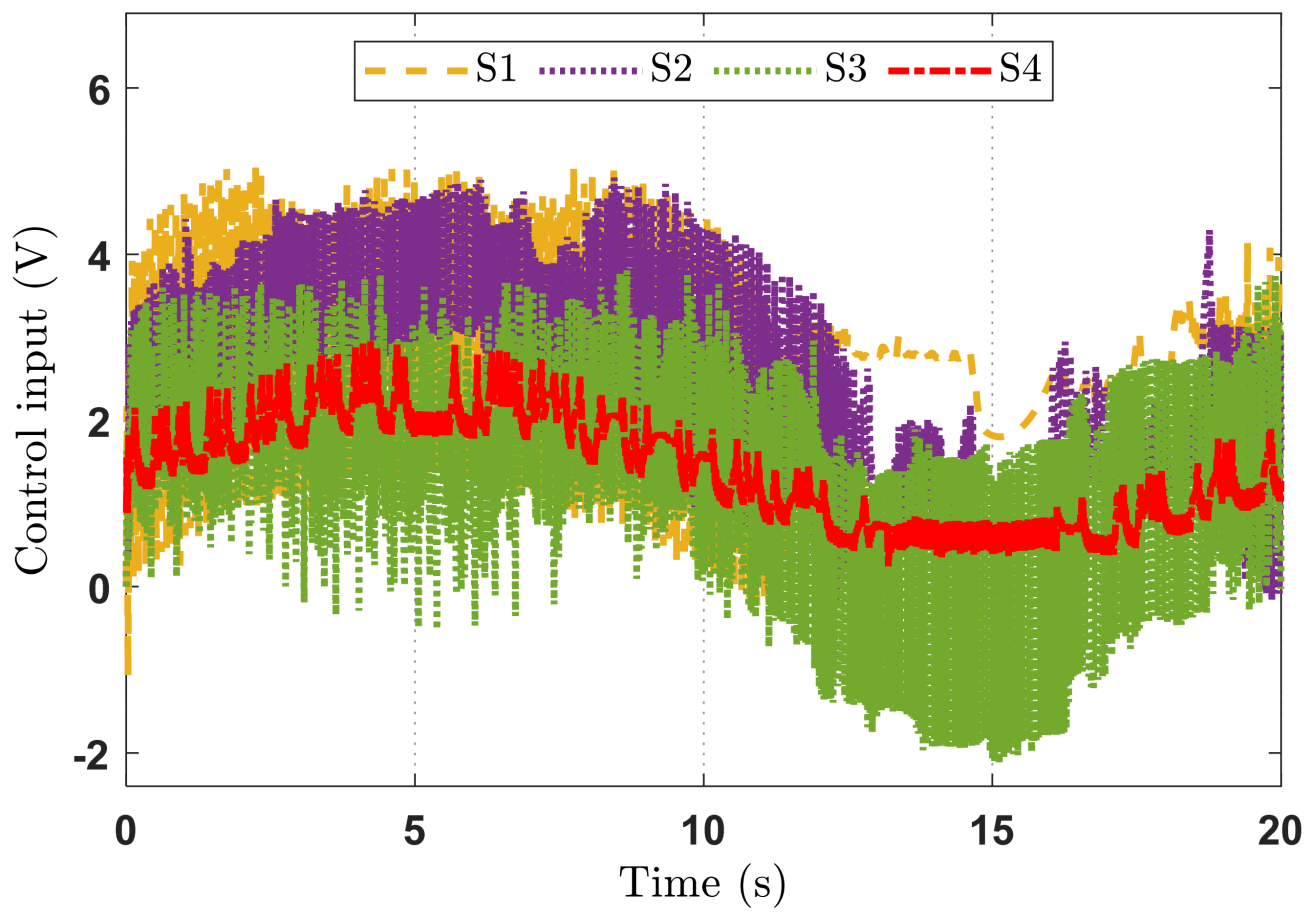
Responses of control inputs under Case 2.

**Figure 11:**
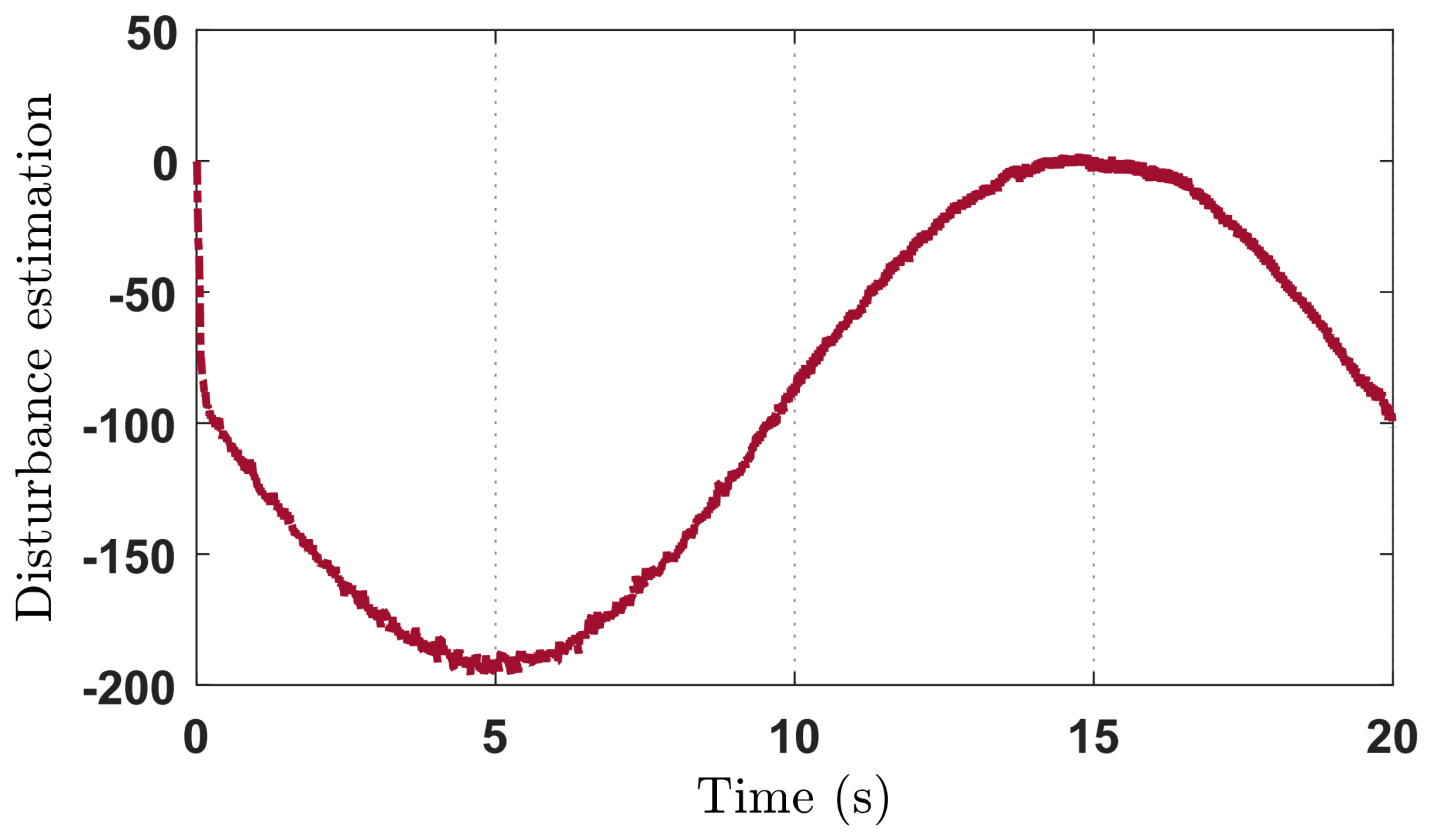
The estimated disturbances of the ESESO under Case 2.

**Figure 12:**
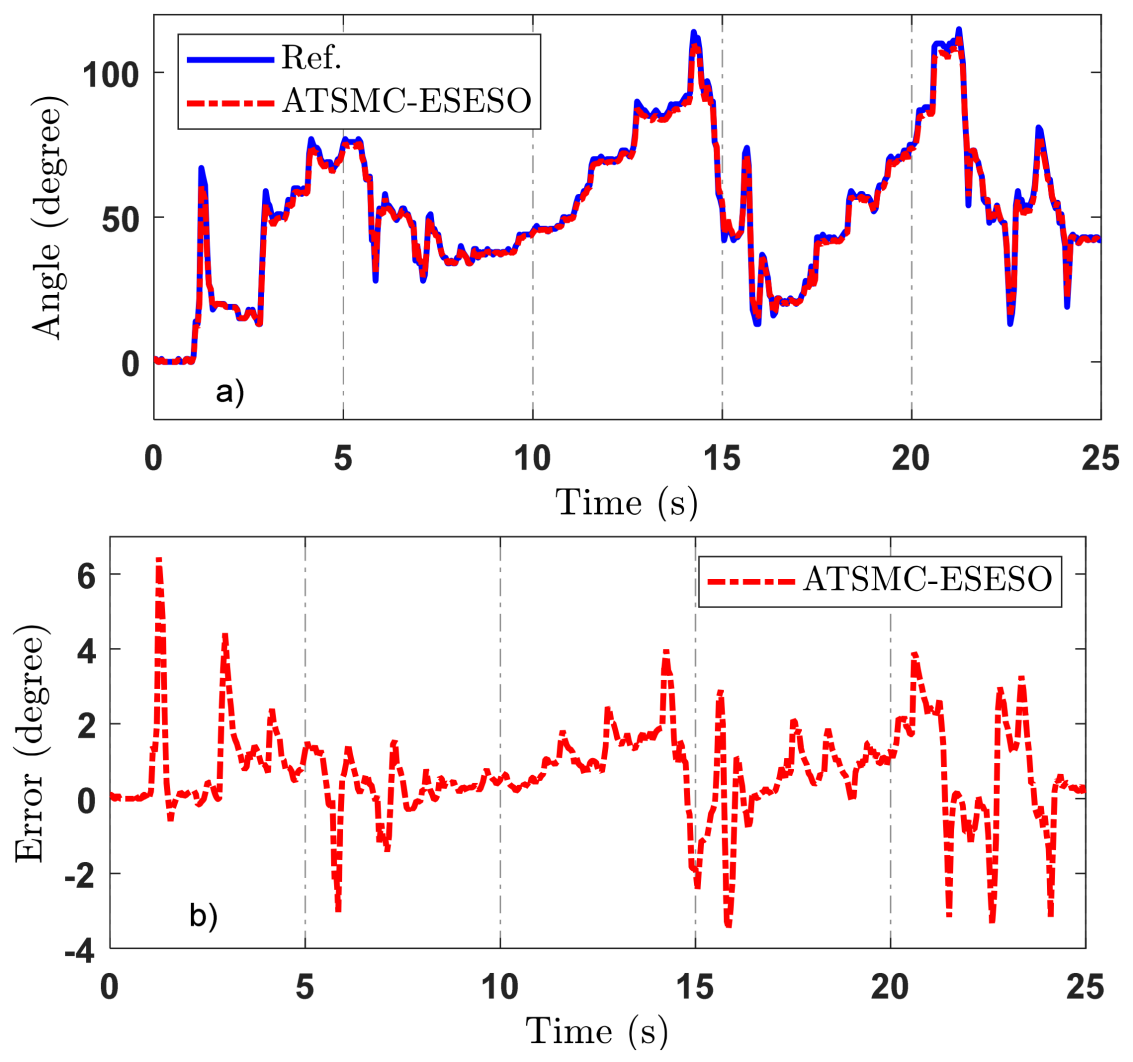

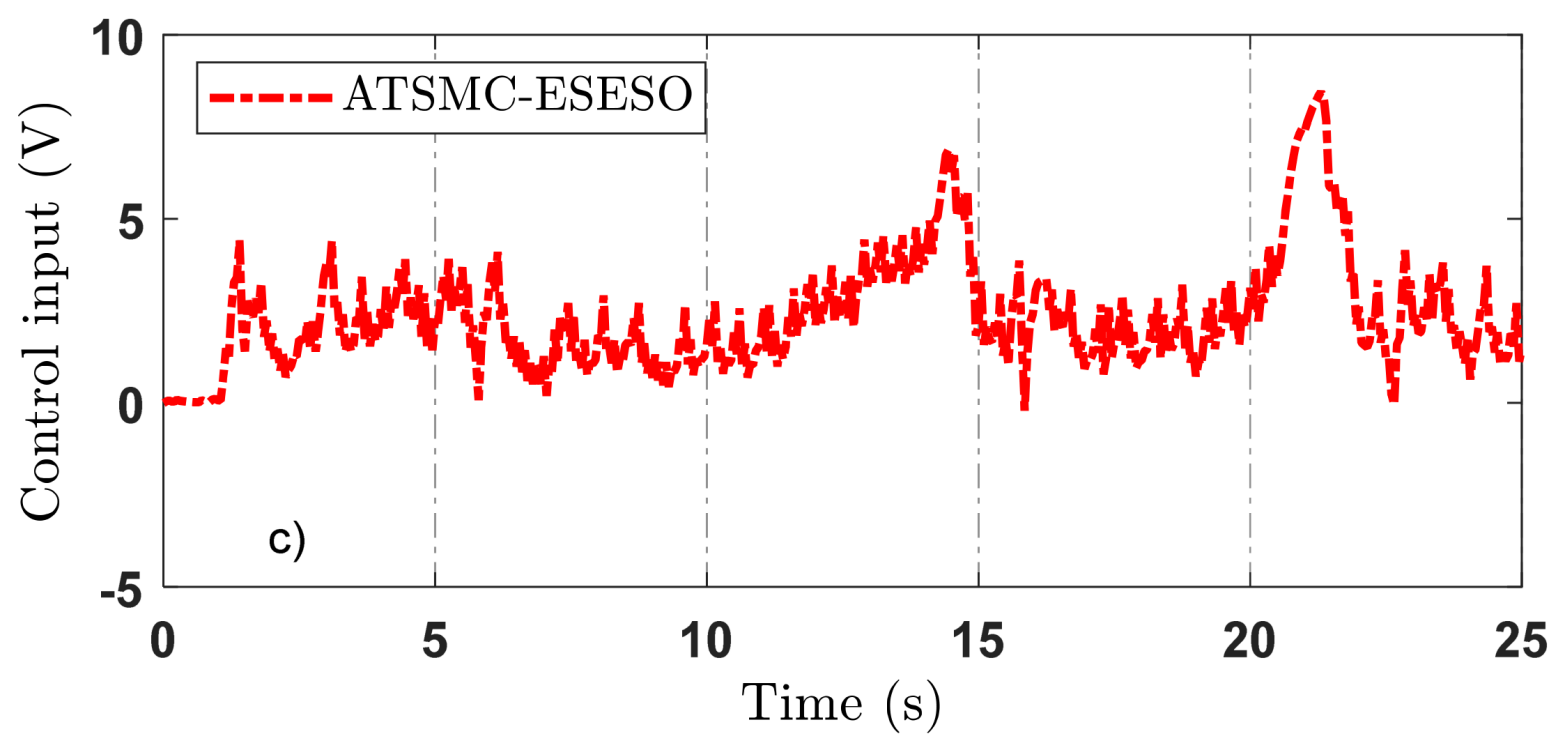
Tracking performance of ATSMC-ESESO under a random reference signal: (**a**) Angle tracking, (**b**) Tracking error, (**c**) Control input.

**Table 1:** Parameters of the EETS [4].

Parameters	Value (Unit)	Parameters	Value (Unit)
*J*	0.098 (kgm^2^)	*K_t_*	0.006 (Nm/A)
*B*	2.4 × 10^−4^ (Nms/rad)	*K_s_*	1.35 (kgm^2^/s^2^)
*R*	2.8 (Ω)	*L*	9 × 10^−4^ (H)
*K_e_*	5 × 10^−3^ (Vs/rad)	*T_f_*	1.5 × 10^−2^ (Nms/rad)

**Table 2:** Component settings of the practical EETS.

Devices	Descriptions	Specification
Throttle valve	Type	Peugeot 3008
	Gearbox	6-speed
	Type of engine	Diesel
	Item Weight	800 g
DC motor	Type	PMDC Motor
	Nominal Voltage	12 [V]
	Dimensions (D × L)	Ø27.9 × 46.5 [mm]
	Motor Regulations	153.225 Rpm/m-Nm
	Torque Constant	16.003 m-Nm/A
DC/DC converter	Type	SmartDriveDuo-30
	Input voltage	10–45 [V]
	Input modes	PWM, Analog
	Dimensions (W × L × H)	81.28 × 101.6 × 42 [mm]
Angle sensor	Type	E6B2-CWZ5B
	Supply power	12–24 [VDC]
	Output phases	A, B, and Z
	Current consumption	80 mA max
	Max. response speed	50 kHz
Battery	Manufacturer	Solite
	Battery Voltage	12 [V]
	Battery type:	Dry, sealed
	Dimensions (L × W × H)	301 × 172 × 220 [mm]
Microprocessor	Type	AVR ARDUINO UNO REV3
	Resolution	8-bits
	Digital I/O	54 (15 PWM)
	Dimensions	68.6 mm × 53.4 mm

**Table 3:** The evaluation of error metrics.

	Case 1		Case 2	
Method	RSE	ME	RSE	ME
S1 (TSMC)	8.339	8.2095	4.3227	3.8826
S2 (ATSMC)	6.4381	6.3177	3.5808	3.0415
S3 (TSMC-ESO)	6.3716	6.1008	1.1593	0.9125
S4 (ATSMC-ESESO)	4.1742	4.1025	0.4735	0.4366

**Table 4:** Evaluation of the chattering phenomenon.

	Case 1		Case 2	
Method	TV	RMSU	TV	RMSU
S1 (TSMC)	2.8497	2.7505	2.682	1.4390
S2 (ATSMC)	2.7625	2.6345	1.8051	1.4390
S3 (TSMC-ESO)	1.6648	1.5239	1.6097	1.2423
S4 (ATSMC-ESESO)	0.4019	0.3701	0.3628	0.2118

## Data Availability

The original contributions presented in this study are included in the article. Further inquiries can be directed to the corresponding author.
